# Opinion on language teacher agency

**DOI:** 10.3389/fpsyg.2022.959013

**Published:** 2022-08-12

**Authors:** Yongli Qin, Ping Wang

**Affiliations:** ^1^School of Foreign Languages, Jiangsu University of Science and Technology, Zhenjiang, China; ^2^Graduate School, Xi'an International Studies University, Xi'an, China

**Keywords:** teacher agency, language teacher, collective agency, teachers' professional development, trans-perspective

The concept of *teacher agency* describes the notion that teachers, as professionals, act as change agents in implementing desired pedagogical changes that sustain relevant curricular reforms (Miller, 2016, p. 352). Recent literature has witnessed a growing interest in detailed research on teacher agency as a “trendy” topic in language teaching and teacher education. Despite much research on teacher agency in professional settings, the concept, especially *language teacher agency*, remains clarified. In this volume, Tao Jian and Gao (Andy) Xuesong, two scholars devoted to teacher education research, elucidate the concept and demonstrate how engagement would promote language teachers' professional development.

This volume comprises six sections besides the introduction section. Following the epistemological order of understanding a construct, Tao and Gao (2021) examine the concept of language teacher agency in terms of the “what”, “why”, and “how” questions in sections 2 to 5. Laying the solid foundation for basic understanding of the construct, they then introduce the concept of *collective agency* and propose a multi-layered model based on an illustrative example of their research project in section 6. They end the book by calling for a *trans*-perspective on language teacher agency for future research.

Teacher agency has been increasingly conceptualized in different educational contexts and teacher education. Tao and Gao reviewed four major theoretical perspectives, social cognitive theory, sociocultural theory, the post-structuralist view, and ecological perspective, to show how the agency is defined and studied under each theoretical framework. The review reveals that scholars, from a social cognitive perspective tend, to view the agency as individuals' intentional acts, while scholars from other traditions emphasize the relationship between actors and contexts more clearly. Research following each line of theory takes on different features in methodology. To be specific, studies on social cognitive perspective are somewhat limited, perhaps because of heavy dependant on survey instruments and statistical analysis. Research on sociocultural perspective usually adopts a qualitative methodology to examine the mediational process. The ecological perspective tends to use case studies to analyze agency as a temporal and situated achievement out of the interplay of “iterational, practical-evaluative, and projective dimensions” (Priestley et al., 2015, p. 34). Post-structuralists adopt a critical discourse analysis methodology based on narratives of all kinds to examine agency from the lens of positioning theory.

Different conceptualizations of teacher agency justify the crucial role in facilitating teachers' professional development. Tao and Gao explore the “why” question and elaborate on why language teacher agency matters in the third section. Drawing on Douglas Fir Group's (2016) framework as a guide for language teacher education, Tan and Gao discuss varied purposes of teacher agency at multiple levels: learning to teach at the individual level, implementing policies at the institutional/national level, and advocating social justice at the societal level. They contribute to the discussion by adding a chronological dimension to the framework to highlight that teacher agency enactment operates throughout a teacher's professional development trajectory. Thus, Tao and Gao clarify that teacher agency works in various contextual conditions and in a teacher's professional career. In particular, they have turned their attention to the group of language teachers and expounded the significance of agentic actions for language teachers to undertake when dealing with the multilingual and multicultural realities in their professional practice and development. This indicates the profound influence of contextual conditions on language teachers' professional practice.

Tao and Gao also acknowledge that teacher agency does not operate alone but interacts with other key constructs in teacher education, such as identity, emotion, belief, and knowledge. In comparison, more attention has been given to exploring teacher agency related to identity. Drawing on the data from their research on language teachers' research engagement in Chinese universities, contrary to the typical focus on language teachers' teaching in primary and secondary schools, Tao and Gao explore the close link between agency and identity. They have found that university language teachers must exercise agency in renegotiating their professional identities and managing research engagement to pursue career opportunities. That is because in many contexts, university language teachers in China are often placed in an ongoing vulnerable position where they are compelled to conduct research and publish in prestigious journals to maintain job security despite their primary duty being to teach language. Although less attention is attached to teacher agency in relation to emotion, beliefs, and knowledge, teacher agency has long penetrated the teaching profession, and more research is expected in the future.

Given the significant role of the agency in shaping teachers' ability and its close links with other constructs in teachers' professional practice and development, Tao and Gao propose two possible approaches to enhance teachers' sense of agency: making changes to contexts and/or promoting teachers' growth. The former is the external factor, which can be realized by building teacher communities of varying sizes, i.e., to create safe and socially supportive groups for teachers, to neutralize the negative impact of contextual constraints on their professional practice. Teachers are the vital internal factors, and only when they make changes by themselves can they develop an enhanced sense of agency. Hence, Tao and Gao advise language teachers to promote teacher agency by creating a reflective space for critical or active reflection and a discursive space to negotiate more favorable professional identities to enhance their sense of agency.

Their previous research uncovers that teachers do not take agentic actions in isolation but often enact them in a socially interactive and dynamic way. Thus, Tao and Gao extend their discussion of teacher agency to the concept of *collective agency*, a seriously under-researched notion in the literature. The joint agency has a root in multiple theorizations of agency, but it is often studied under a sociocultural approach. Based on their research project on the multilingual research team, Tao and Gao propose a multi-layered framework to illustrate further how collective agency emerges. Their findings demonstrate that the joint agency operates at multiple levels, including between individuals, within subgroups, and in a team as a whole. These levels interact to produce a sustained sense of collective agency among language teachers, thus enabling them to transform from individual actors to supportive and collaborative community members. They can seek and lend intellectual “resources” and emotional support.

Tao and Gao advance a trans-perspective of agency for future research at the end of the article. Guided by this trans-perspective on language teacher agency, more prospective studies are expected to integrate theoretical frameworks from other disciplines and/or experiments with new methodologies from different fields to uncover more aspects of the agency. We argue that this thought-provoking idea fits with the current trend of transdisciplinary research on applied linguistics and conforms to the transnational, transcultural, and translingual settings where language teachers live and work.

To sum up, in this small-sized volume, Tao and Gao have presented a panorama of language teacher agency for readers with a brief but thorough exploration of the concept in terms of what agency is, why it matters, what it does, and how it can be approached in research and practice. Their comprehensive introduction makes the concept more accessible for readers. The uniqueness of this book lies in that apart from the multi-layered model and the *trans*-perspective of agency they put forward, the examples they used are different from the majority of existing studies on language teachers' professional practices. For one thing, most existing studies focus on primary and secondary schools, but Tan and Gao explore language teacher agency at the tertiary level. Previous studies have focused on language teachers' teaching, while their projects are primarily concerned with language teachers' research engagement, an important but under-examined aspect of language teachers' multifaceted professional practices (p. 2). In this way, Tao and Gao extend the research scope of teacher agency and shed light on future research by applying the concept to a more barely studied aspect of language teachers' professional practices.

As language teachers, we benefit a lot from reading. This book has informed us of language teacher agency and inspired us to exercise the collective agency manifested in seeking academic and emotional support in a community for professional development. In short, this book achieves the purpose of making readers better understand the concept of teacher agency. However, if some suggestions should be made, we suggest that it is better to provide a reference list after each section, which will help readers read the given topic further. Despite this flaw, this is still a good reference book for teacher educators, practitioners, and policymakers who will develop a critical and essential understanding of the construct after reading.

## Author contributions

YQ wrote the manuscript. PW revised the manuscript. All authors listed have made a substantial, direct, and intellectual contribution to the work and approved it for publication.

## Funding

This research was supported by the Social Science Foundation of Jiangsu Province (Grant No. 21YYB011) and Xi'an International Studies University (Grant No. BSZA2019002).

## Conflict of interest

The authors declare that the research was conducted in the absence of any commercial or financial relationships that could be construed as a potential conflict of interest.

## Publisher's note

All claims expressed in this article are solely those of the authors and do not necessarily represent those of their affiliated organizations, or those of the publisher, the editors and the reviewers. Any product that may be evaluated in this article, or claim that may be made by its manufacturer, is not guaranteed or endorsed by the publisher.
